# Alloferon and Zanamivir Show Effective Antiviral Activity against Influenza A Virus (H1N1) Infection In Vitro and In Vivo

**DOI:** 10.3390/ijms24010678

**Published:** 2022-12-30

**Authors:** Dahae Lee, Hyejung Jo, Yoojin Jang, Suhyun Bae, Tomoyo Agura, Dongmin Kang, Minsoo Kang, Yuri Kim, Nam-Hyuk Cho, Yejin Kim, Jae Seung Kang

**Affiliations:** 1 Laboratory of Vitamin C and Antioxidant Immunology, Department of Anatomy and Cell Biology, Seoul National University College of Medicine, Seoul 03080, Republic of Korea; 2 Department of Psychological and Brain Sciences, College of Arts and Sciences, Boston University, Boston, MA 02215, USA; 3 Korea International School in Pangyo, Seongnam-si 13543, Republic of Korea; 4 Department of Biomedical Sciences, Seoul National University College of Medicine, Seoul 03080, Republic of Korea; 5 Department of Microbiology and Immunology, Seoul National University College of Medicine, Seoul 03080, Republic of Korea; 6 Institute of Endemic Diseases, Seoul National University College of Medicine, Seoul 03080, Republic of Korea; 7 Seoul National University Bundang Hospital, Seongnam-si 13620, Republic of Korea; 8 Medical Research Center, Institute of Allergy and Clinical Immunology, Seoul National University, Seoul 03080, Republic of Korea

**Keywords:** alloferon, zanamivir, influenza virus A/H1N1, lung inflammation

## Abstract

The use of vaccines is the most effective and reliable method for the prevention of viral infections. However, research on evaluation of effective therapeutic agents for use in treatment after infection is necessary. Zanamivir was administered through inhalation for treatment of pandemic influenza A/H1N1 in 2009. However, the emergence of drug-resistant strains can occur rapidly. Alloferon, an immunomodulatory drug developed as an NK cell activator, exerts antiviral effects against various viruses, particularly influenza viruses. Therefore, alloferon and zanamivir were administered in combination in an effort to improve the antiviral effect of zanamivir by reducing H1N1 resistance. First, we confirmed that administration of combined treatment would result in effective inhibition of viral proliferation in MDCK and A549 cells infected with H1N1. Production of IL-6 and MIP-1α in these cells and the activity of p38 MAPK and c-*Jun* that are increased by H1N1 were inhibited by combined treatment. Mice were then infected intranasally with H1N1, and examination of the antiviral efficacy of the alloferon/zanamivir combination was performed. The results showed that combined treatment after infection with H1N1 prevented weight loss, increased the survival rate, and improved lung fibrosis. Combined treatment also resulted in reduced infiltration of neutrophils and macrophages into the lungs. Combined treatment effectively inhibited the activity of p38 MAPK and c-*Jun* in lung tissue, which was increased by infection with H1N1. Therefore, the combination of alloferon/zanamivir effectively prevents the development of H1N1-mediated inflammation in the lungs by inhibiting the production of inflammatory mediators and migration of inflammatory cells into lung tissue.

## 1. Introduction

With an increased incidence of viral infections including SARS-CoV2 worldwide, the research and development of vaccines for the prevention of viral infection has also shown a recent increase [1,2]. Vaccines are known to be effective in the prevention of viral infection [3,4]; however, with regard to viral infectious diseases caused by avoidance of immune responses induced through vaccination, the development of therapies for the treatment of viral infection is important. Therefore, this study was conducted in order to establish an effective method for enhancing the efficacy of zanamivir, which is used for the treatment of viral infection after infection with the influenza virus.

Influenza, a seasonal infectious disease, is responsible for a significant number of deaths reported annually worldwide [5,6,7]. The influenza virus, a single-stranded RNA virus belonging to the Orthomyxoviridae family, is classified according to four types: Type A, Type B, Type C, and Type D. Of these, the H1N1 and H3N2 subtypes (both influenza A viruses) are primary causes of acute severe respiratory diseases in humans [8,9]. Influenza A viruses pose a threat to the health of humans and animals due to their high rates of transmission, leading to high mortality and morbidity [10,11]; in addition to humans, acute respiratory infections caused by the virus can also affect birds and pigs [12,13,14]. Two types of antigens are located on the surface of the influenza A virus: hemagglutinin (HA) and neuraminidase (NA) [15,16]. The importance of HA during infection of host cells has been reported, and NA is utilized by viruses for infection of other cells following transcription of viral RNA [17,18]. The therapeutic agents developed to date for treatment of influenza A virus infection (e.g., oseltamivir and zanamivir) inhibit the function of NA [19,20].

Alloferon, a naturally occurring immunomodulatory peptide, detected in the blood and lymph nodes of blow flies (Calliphora vicina) infected with bacteria, has been reported [21,22]. It consists of 13 amino acids: HGVSGHGQHGVHG [22,23,24]. Recent studies have demonstrated its involvement in control of acute inflammatory responses in epithelial cells in the skin and cornea, as well as chronic inflammatory responses in rheumatoid arthritis and asthma [25]. Its antiviral functions also regulate infection and recurrence of herpes simplex virus [21,26]. Mediation of the antiviral and anti-tumor efficacy appears to occur via an increase in the activity of human NK cells and production of interferon [27]. In fact, conduct of clinical studies on the antiviral effects of alloferon against HCV and influenza virus is underway in Russia [28,29].

The development of amantadine, rimantadine, oseltamivir, and zanamivir as antiviral drugs for the treatment of H1N1 and H3N2 has been reported [30,31]. However, globally, more than 600,000 people die each year from the virus, especially when the seasons change [32,33,34,35]. Infection with influenza A virus can also affect animals, thus, effective prevention of transmission between animals and humans, and development of effective drugs and treatments is necessary [36,37]. Of the antiviral drugs used for prevention of influenza A virus infection, amantadine and rimantadine are not currently in use due to the rapid development of resistance [38,39]. Oseltamivir and zanamivir, which bind to the NA receptor in host cells to prevent release of viruses from infected cells by NA, are used most often [40,41,42]. However, the side effects of oseltamivir, including nausea, vomiting, headache, and gastrointestinal disorders, as well as psychological disorders such as delirium, convulsions, and hallucinations, particularly in adolescents, have been reported [36,43,44]. In addition, the development of drug resistance is more rapid compared with that for zanamivir.

Zanamivir is inhaled into the nasal cavity, therefore, because most of the drug is delivered to the lungs there are fewer systemic side effects compared with oseltamivir [45,46,47]; however, there is a disadvantage in that the rate of absorption is lower than that of oseltamivir, so that the drug shows a tendency to be less effective. It is known to bind to the active site of the neuraminidase of the influenza virus to prevent the spread of the virus and to inhibit the replication of the virus by itself [48,49]. According to a paper published in PNAS by Sergey et al. in 2002, it was reported that the production of IFN was increased in both subcutaneous injection and intranasal administration of alloferon, and that intranasal administration of alloferon was more effective than subcutaneous injection [28]. Therefore, in this study, we attempted to determine whether administration of a combination of zanamivir and alloferon can compensate for the weaknesses of the only zanamivir when used for treatment of influenza A infection.

## 2. Results

### 2.1. Alloferon Increases the Antiviral Effect of Zanamivir against H1N1 Infection

**Figure 1:**
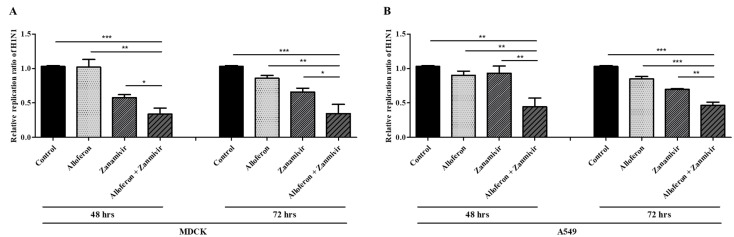
Alloferon increased the antiviral efficacy of zanamivir after infection with H1N1. Quantitative real-time PCR analysis of the levels of influenza virus (normalized to GAPDH) was performed in the presence or absence of alloferon (0.5 μg/mL) and zanamivir (35 μg/mL). Briefly, cells were infected with influenza A virus (MOI 0.01), extraction of total RNA was performed, and RT-PCR was performed using primers specific for H1N1. The amount of virus in treated cells is shown relative to that in control cells (cells infected with virus but not treated). Results are representative of more than three independent experiments. Control: Virus only (**A**) MDCK (**B**) A549 * *p* < 0.1, ** *p* < 0.01, *** *p* < 0.001, NS; not significant.

First, real-time RT-PCR was performed in order to assess the effect of alloferon and zanamivir on viral proliferation in H1N1-infected Madin-Darby canine kidney (MDCK), A549, adenocarcinomic human alveolar basal epithelial cell line and Calu-3, human lung adenocarcinoma cell line. As shown in Figure 1, when combined with zanamivir, alloferon showed more effective suppression of viral replication in both cell lines, A549 and MDCK, compared with control and a group treated with either drug alone. The suppressive effect was observed from 48 h post treatment and lasted for up to 72 h. A more efficient effect on the suppression of virus replication was observed in A549 cells compared with MDCK cells. In Calu-3 cells, the suppressive effect mediated by treatment with alloferon/zanamivir was also apparent at 48 h (see Supplementary Figure S1).

### 2.2. Combination Alloferon/Zanamivir Suppresses Production of IL-6 and MIP-1α Induced by H1N1 Infection In Vitro

**Figure 2:**
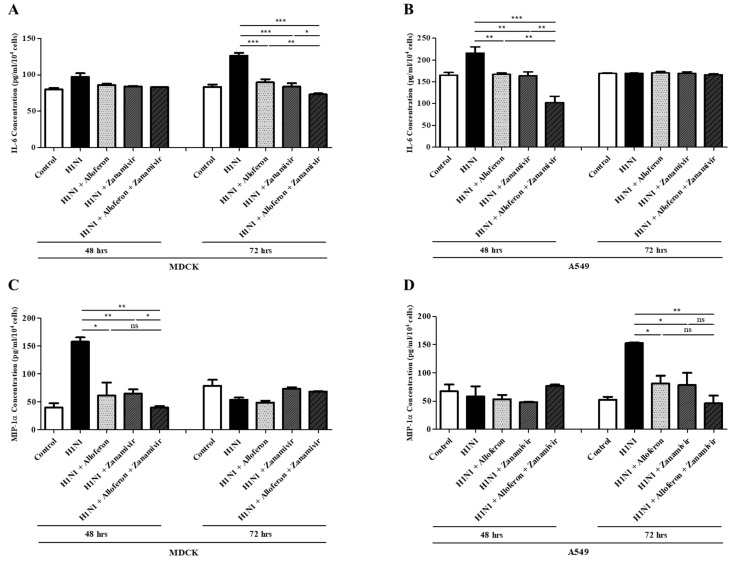
Combined treatment with alloferon and zanamivir suppressed production of IL-6 and MIP-1α in H1N1-infected cells MDCK and A549 in vitro. At 48 and 72 h post-H1N1 infection, culture supernatants were collected and measurement of the levels of IL-6 and MIP-1α in the presence or absence of alloferon (0.5 μg/mL) and/or zanamivir (35 μg/mL) was performed using an enzyme-linked immunosorbent assay (ELISA) as described in the Section 4. Results are representative of more than three independent experiments. Control: Virus uninfected cells (**A**,**C**) MDCK (**B**,**D**) A549 * *p* < 0.1, ** *p* < 0.01, *** *p* < 0.001, NS; no significant.

Infection with influenza A virus induces the production of IL-6 and MIP-1α, which are involved in development of sepsis and induction of the cytokine storm in the lung [50,51,52,53]. Therefore, the question of whether combined treatment with alloferon and zanamivir can suppress production of IL-6 and MIP-1α after infection with H1N1 was examined. The results showed that the production of IL-6 in MDCK cells increased at 72 h post infection with H1N1, while increased production of MIP-1α was observed at 48 h post infection; however, effective suppression of both was achieved by combined treatment with alloferon and zanamivir (Figure 2A,C). Regarding A549, increased production of IL-6 was observed at 48 h after infection with H1N1, and an increased production of MIP-1α was observed at 72 h; combined treatment with alloferon and zanamivir resulted in suppression of both (Figure 2B,D).

### 2.3. The Antiviral Effects of Combined Treatment with Alloferon and Zanamivir Are Mediated by Inhibition of the p38MAPK and JNK Signaling Pathways

**Figure 3:**
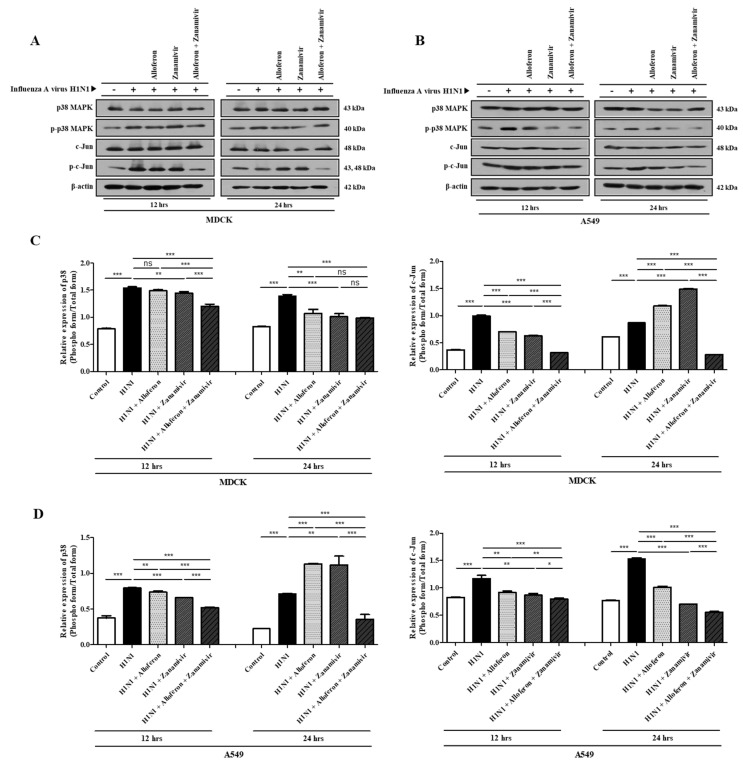
The antiviral effects of combined treatment with alloferon (0.5 μg/mL) and zanamivir (35 μg/mL) are mediated by inhibition of the p38 MAPK and JNK signaling pathways. MDCK (**A**) and A549 (**B**) cells were cultured in the presence or absence of alloferon and/or zanamivir for 12 and 24 h. Cell lysates were prepared. and western blot analysis was performed for detection of changes in activation of p38 MAPK and JNK, as described in the Materials and Methods Section 4. Results are representative of more than three independent experiments. (**C**,**D**) Calculation of the relative expression of the phospho form/total form was performed using Image J software. * *p* < 0.1, ** *p* < 0.01, *** *p* < 0.001, NS; no significant.

Activation of p38 mitogen activated protein kinase (MAPK) and Jun-amino-terminal kinase (JNK) in virus infected cells in the presence or absence of alloferon (0.5 μg/mL) and/or zanamivir (35 μg/mL) was examined in order to confirm the signaling pathways involved in the antiviral effects of combined treatment with alloferon and zanamivir. As shown in Figure 3A,B, infection of MDCK and A549 cells with H1N1 resulted in increased phosphorylation of p38 MAPK and JNK, which was suppressed by both drugs individually and in combination, although combination treatment was more effective. Regarding P38MAPK, its phosphorylated form appeared to show a decrease in the group treated only with Zanamivir, however, a decrease in the total form of p38MAPK was also observed. Therefore, no difference in the ratio was observed when phosphorylated p38MAPK was divided by total p38MAPK. An analysis of all bands was performed using Image J software in order to clarify the results, as shown in Supplementary Figure S6.

### 2.4. Combined Treatment with Alloferon and Zanamivir Inhibits Viral Replication and Increases Survival of Mice Infected with H1N1

An experiment was performed to determine the optimal amount of alloferon and zanamivir prior to conduct of an experiment to confirm the antiviral effect. According to our previous report on the effect of alloferon [21,23,24,25,54,55], 0.5–2 μg/mL was used for examination of the antiviral efficacy or immune enhancement function. Determination of a dose of 35 μg/mL of zanamivir was based on conversion of the amount administered for the treatment of patients with influenza virus infection into a weight of mice. As a result, alloferon, 0.5 and 1 μg/mL and zanamivir 35 μg/mL and 70 μg/mL were established for study on the combined treatment of alloferon and zanamivir, and a pilot study was conducted, as shown in Supplementary Figure S3. According to the results shown in Supplementary Figure S3, the optimal concentration for the drug combination was determined as 0.5 μg alloferon and 35 μg zanamivir. The results confirmed that there was no cytotoxicity at that concentration (Supplementary Figure S4). Regarding in vivo toxicity and safety, there was no acute toxicity in mice (up to 6000 mg/kg), rat (up to 5000 mg/kg) and dog (up to 20 mg/kg) as well as no embryo-toxic or teratogenic effect in rat (up to 15 mg/kg). In addition, no special toxicity was observed in phase 1 clinical studies in which 0.1, 1, 5, and 10 mg of alloferon were administered subcutaneous in 40 healthy people. In a phase 2 clinical study of patients with recurrent genital herpes and acute hepatitis B virus infection, it was observed that it was effective in controlling diseases caused by viruses. In our previous report [27], 25–50 μg/mL was mainly used in experiments to confirm in vivo anti-cancer efficacy, but 0.5 μg/mL of alloferon were used in this experiment. It is quietly lower concentration than the concentration in the experiment for anti-cancer experiment.

**Figure 4:**
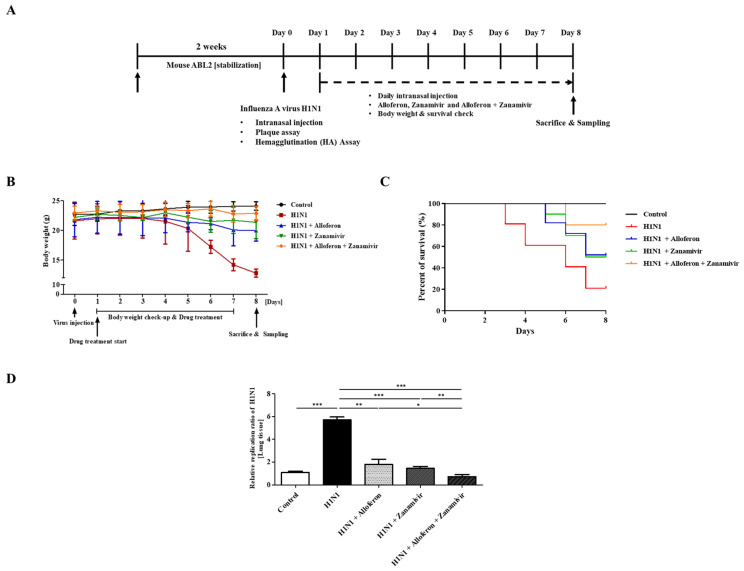
Combined treatment with alloferon (0.5 μg/mL) and zanamivir (35 μg/mL) increased the survival rate of mice infected with H1N1 by inhibiting replication of the virus. (**A**) Intranasal infection of mice with H1N1 (1:32 diluted in saline) was performed after measurement of the effective viral titer using an HA assay (Supplementary Figure S5). At 24 h post-virus infection, a daily dose of alloferon and/or zanamivir was administered intranasally in mice (*n* = 30/group) for seven days. (**B**) Body weight and (**C**) survival were monitored for an additional eight days. (**D**) Total RNA was extracted from lung tissues and an analysis of viral replication was performed using real-time PCR as described in the Section 4. Results are representative of more than three independent experiments. * *p* < 0.1, ** *p* < 0.01, *** *p* < 0.001.

Based on the in vitro results from MDCK and A549 cells, the antiviral effects of alloferon and zanamivir in mice infected with H1N1 were then examined using the experimental scheme shown in Figure 4A. H1N1 was administered intranasally to mice after performance of an HA assay for measurement of the effective viral titer (Figure 4A). On Day 4 post virus infection, a decrease in weight was observed in untreated virus inoculated mice, which was effectively rescued by combined treatment with alloferon and zanamivir (Figure 4B). In addition, the survival rate of infected mice began to show a decline at three days post inoculation with virus; however, the rates showed marked improvement after combined treatment with alloferon and zanamivir (Figure 4C). Real-time RT-PCR was also performed in order to examine replication of H1N1 in the lungs of mice treated with (or without) alloferon and/or zanamivir (Figure 4D), as described in the Section 4. As in MDCK and A549 cells, combined treatment with alloferon and zanamivir resulted in markedly inhibited proliferation of H1N1.

### 2.5. Combined Treatment with Alloferon and Zanamivir Suppresses Production of IL-6 and MIP-1α Induced by H1N1 Infection In Vivo

**Figure 5:**
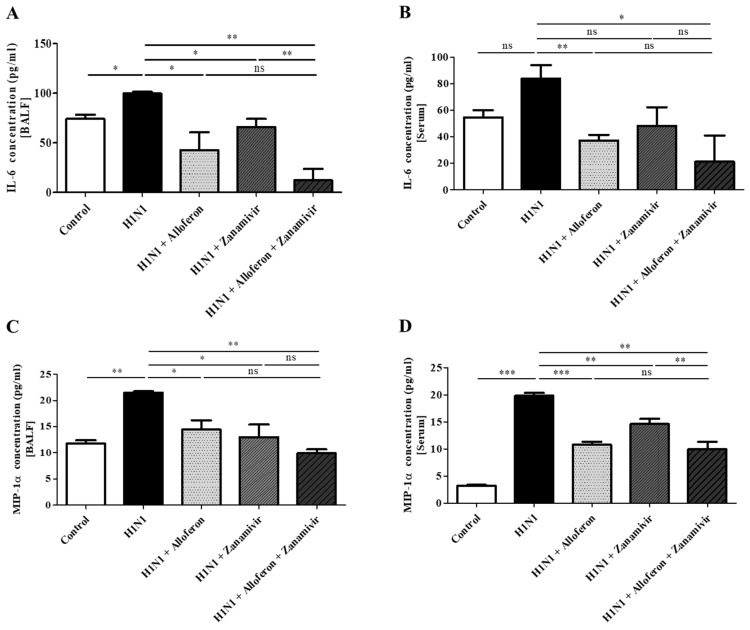
Combined treatment with alloferon (0.5 μg/mL) and zanamivir (35 μg/mL) suppressed H1N1-mediated production of IL-6 and MIP-1α in vivo. BALF (bronchoalveolar lavage fluid) (**A**,**C**) and serum (**B**,**D**) were collected, and ELISA was performed for measurement of the levels of IL-6 and MIP-1α as described in the Section 4. Reading of plates was performed using a microplate reader at 450 nm. Results are representative of more than three independent experiments. * *p* < 0.1, ** *p* < 0.01, *** *p* < 0.001, NS; no significant.

An examination of the levels of these molecules in serum and BALF from mice infected with H1N1 was performed. As described above, we demonstrated that production of IL-6 and MIP-1α in MDCK and A549 cells was suppressed by combination of alloferon/zanamivir (Figure 2). Similar to the in vitro results, combined treatment with alloferon and zanamivir resulted in suppression of the H1N1-mediated increase in the amounts of IL-6 and MIP-1α in Broncho alveolar lavage fluid (BALF) (Figure 5A,C); however, no change in the levels of IL-6 and MIP-1α in serum were observed (Figure 5B,D).

### 2.6. Pathological Changes in the Lungs of H1N1-Infected Mice Are Prevented by Combined Treatment with Alloferon and Zanamivir

**Figure 6:**
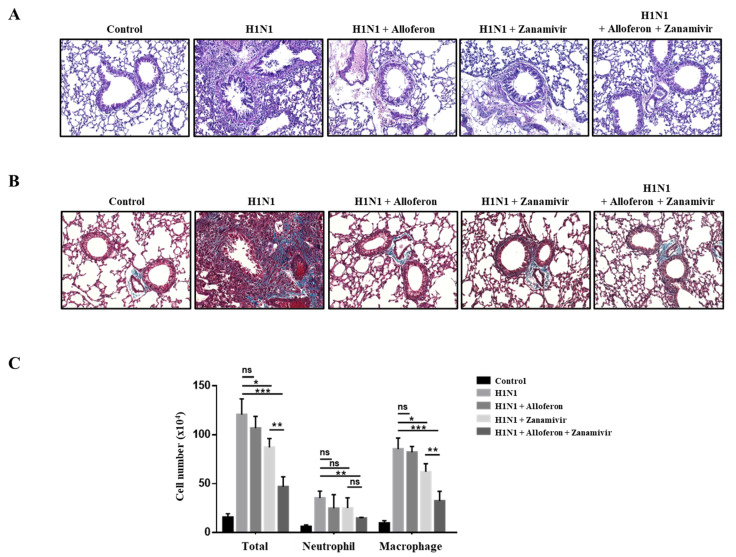
Pathological changes in the lung induced by infection with H1N1 were effectively prevented by combined treatment with alloferon (0.5 μg/mL) and zanamivir (35 μg/mL). Lung tissues were collected from mice and embedded in paraffin. Sectioning (4 µm thick) and staining of tissues were performed as described in the Section 4. Cells were collected by flushing the lungs with ice-cold PBS containing EDTA (0.03%), and cell numbers were counted. (**A**) Hematoxylin & Eosin Staining. (**B**) Masson’s Trichrome staining. (**C**) Number of infiltrating inflammatory cells in the BALF. Results are representative of more than three independent experiments. * *p < 0.1,* ** *p* < 0.01, *** *p* < 0.001, NS; no significant. Scale bar, 100 μm.

Following the harvest of lung tissues from mice, histological examination was performed in order to evaluate both pathological changes and cytokine production, caused by infection with H1N1, and to determine whether combined treatment with alloferon and zanamivir can inhibit these pathological changes. As shown in Figure 6A, widespread inflammatory lesions were detected in the lungs of mice infected with H1N1. However, treatment with alloferon and zanamivir effectively prevented these pathological changes, particularly when used in combination (Figure 6). Further analysis was performed using Masson’s Trichrome staining in order to examine fibrotic changes in lung tissue caused by infection with H1N1. Severe fibrosis caused by H1N1 was detected in lungs, which was effectively prevented by combined treatment with alloferon and zanamivir (Figure 6B). The effect of combined treatment with alloferon and zanamivir on the number of immune cells, particularly neutrophils and macrophages, in the BALF of H1N1-infected mice was also examined. Combined treatment with alloferon and zanamivir resulted in a decrease of the total number of immune cells in BALF; however, the number of macrophages and neutrophils showed a particularly significant decline (Figure 6C).

### 2.7. Combined Treatment with Alloferon and Zanamivir Inhibits H1N1-Induced Activation of p38 MAPK and JNK Signaling Pathways in Lung Tissue

**Figure 7:**
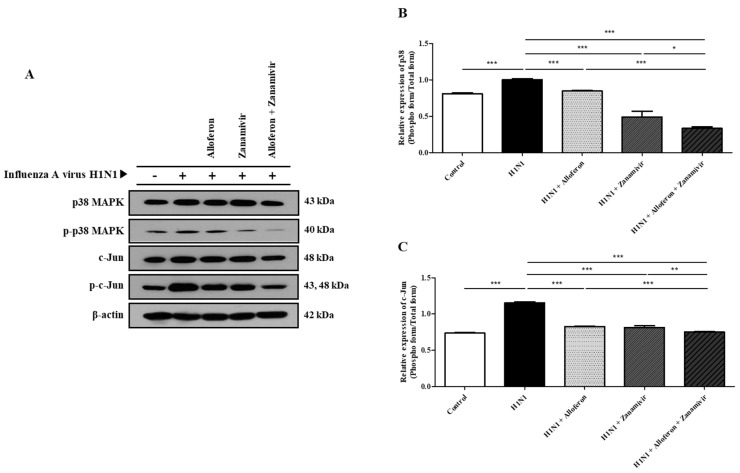
Combined treatment with alloferon (0.5 μg/mL) and zanamivir (35 μg/mL) inhibited H1N1-induced activation of the p38 MAPK and JNK signaling pathways in mouse lung tissue. (**A**) Lung tissues were collected after sacrifice, lysates were prepared in RIPA buffer, and western blot analysis was then performed for detection of p38 MAPK and JNK as described in the Section 4. Results are representative of more than three independent experiments. (**B**,**C**) Calculation of the relative intensity of the phospho form/total form was performed using Image J software. * *p <* 0.1 (The *p* < 0.1 statistical implications are results for determining tendency only), ** *p* < 0.01, *** *p* < 0.001, NS; no significant.

As shown in Figure 3, combined treatment with alloferon and zanamivir inhibited H1N1-induced activation of the p38 MAPK and JNK signaling pathways in MDCK and A549 cells. Therefore, we attempted to determine whether the same result would be obtained in vivo. The results showed that combined treatment with alloferon and zanamivir resulted in significant suppression of H1N1-induced activation of p38 MAPK and JNK in lung tissue (Figure 7). Analysis of WB images was also performed using Image J software in order to clarify the results, as shown in Supplementary Figure S6.

## 3. Discussion

Zanamivir is known to bind to the active site of the neuraminidase of the influenza virus to prevent the spread of the virus and to inhibit the replication of the virus by itself [20,56]. According to a paper published in PNAS by Sergey et al. in 2002, it was reported that the production of IFN was increased in both subcutaneous injection and intranasal administration, and that intranasal administration was more effective than subcutaneous injection [28]. In addition, we have already found that IFN production was increased by alloferon in our previous study [27]. Therefore, it is thought that it will be more effective if alloferon and interferon are used together, since alloferon indirectly shows it anti-viral effect through the increase of IFN production.

The study of infectious diseases and their causes has long been an important area of research in the medical field. The introduction and adoption of a variety of vaccines in different countries around the world has enabled control of viral infection. Two classes of antiviral drugs are currently approved for treatment of influenza in humans; adamantanes (amantadine and rimantadine) and neuraminidase inhibitors (oseltamivir, zanamivir, and peramivir) [57,58,59]. Many studies have reported on resistance to these drugs [60,61,62], and development of chemical conjugates as treatments has been reported [63,64]. Conduct of research on new approaches such as this one is underway, however, no studies on the synergistic effects have been reported. Therefore, our study examined the effect of combination of zanamivir, a drug approved for treatment of influenza viruses, and alloferon, which has several immune effects and is much more effective than a single agent against influenza viruses. Alloferon was developed as an immunomodulatory agent; however, it also has anti-tumor [54], anti-inflammatory [23],and anti-viral [55] efficacy, particularly against influenza A virus [24]. Zanamivir was developed for the treatment of infection with influenza A virus. Compared with other antiviral agents, it has a higher safety profile, but less antiviral efficacy when used for treatment of infection with influenza A virus [65,66].

MDCK, A549, and Calu-3 cells were used in this study to assess the effects of combined treatment with alloferon and zanamivir against infection with influenza A virus. The results obtained from experiments using MDCK and A549 cells suggest that the extent of effective suppression of viral replication with combined treatment is significantly greater than that obtained with use of either alloferon or zanamivir alone. Although A549 and Calu-3 are known lung adenocarcinoma cell lines, we focused more attention on A549 because it is derived from type II alveolar epithelial cells, while Calu-3 is derived from the submucosal layers of bronchial airways. This is because in the case of animal experiments, the focus was on identifying changes in the lungs, rather than changes in the bronchial tubes. Therefore, data acquired from A549 cells was compared with data from MDCK cells. In addition to the in vitro studies using MDCK and A549 cells, our findings confirmed that combined treatment with alloferon and zanamivir (1) prevented weight loss in mice infected with H1N1; (2) increased survival rates post-infection; (3) inhibited viral replication in lung tissue; and (4) inhibited inflammatory responses and pulmonary fibrosis. Considering these findings, the data suggested the usefulness of alloferon as an adjuvant capable of enhancing the antiviral efficacy of zanamivir. In our experiments, alloferon along with zanamivir was administered directly into the nasal cavity of mice infected with H1N1. As a result, alloferon effectively induced an *increase* of the efficacy of zanamivir, which is safe, but less effective than other drugs due to its local action on respiratory tissues and the fact that it does not spread systemically. In general, a nasal spray is used for delivery of drugs administered into the nasal cavity, so that a smaller amount of drug is delivered to the target lesion compared with oral or intravenous administration. In an effort to compensate for this, we are also conducting research on formulations that involve combination of alloferon and zanamivir with drug carriers capable of more effective transfer of drugs to target tissues. These studies are expected to provide evidence that the combination of alloferon and other drugs is an effective option for treatment of respiratory diseases.

The Th1 immune response is suppressed and the Th2 immune response is boosted in humans and mice infected with influenza A virus [67]. This explains the symptoms that are observed with proliferation of the influenza A virus. Among the Th2 cytokines, IL-6 can trigger a “cytokine storm” (known as cytokine release syndrome), as well as macrophage activation syndrome [68,69]. As shown in Figure 2, increased production of IL-6 in MDCK and A549 cells was observed after infection with H1N1; however, production of IL-6 showed a marked decreased after treatment with combination alloferon/zanamivir that was significantly greater than that after treatment with alloferon or zanamivir alone. In addition, the amount of IL-6 in the BALF of virus-infected mice showed a significant decrease after treatment with combination alloferon/zanamivir (Figure 5). This finding indicates that combination alloferon/zanamivir might be an effective treatment for H1N1-induced inflammation of lungs. Like IL-6, increased production of MIP-1α was observed in MDCK and A549 cells, as in H1N1-infected mice; however, levels of H1N1 infection showed a significant decrease after treatment with combination alloferon/zanamivir (Figure 2 and Figure 5).

Increased levels of MIP-1α (also known as CCL3), a chemokine in inflammatory diseases such as rheumatoid arthritis, and in tumors such as multiple myeloma, have been reported [70,71]. It is produced primarily by macrophages, but also by other types of lymphocytes including T cells, B cells, and NK cells [72,73]. Synthesis of MIP-1α by non-hematopoietic cells such as osteoblasts and inflamed epithelial cells occurs upon stimulation with viruses [38,73]. During the inflammatory process, macrophages are recruited to sites where tissue damage has occurred, where they initiate the process of regeneration and repair [74,75]. An increase in the levels of MIP-1α occurs upon viral infection, particularly after infection with respiratory viruses such as Respiratory *Syncytial* Virus (RSV) and influenza virus. For example, Respiratory *Syncytial* Virus (RSV) causes an increase in the activity of neutrophils in lung tissue, leading to severe damage [76,77]. In addition, alleviation of pneumonia in MIP-1α-deficient mice infected with the influenza virus has been reported [78]. Increased levels of MIP-1α have also been reported in patients with asthma and pulmonary fibrosis, and in the alveolar fluid of patients with acute respiratory distress syndrome (ARDS) [78,79]. The results of this study showed that the production of IL-6 and MIP-1a in the BALF of mice infected with H1N1 was inhibited by combination alloferon/zanamivir (Figure 5). Thus, combination alloferon/zanamivir might also be an effective treatment for inflammatory responses caused by infection with respiratory viruses, as well as for regulation of inflammatory responses in the lung mediated by IL-6 and MIP-1a.

Activation of p38 MAPK has a close association with replication of influenza viruses; suppression of the activity of p38 MAPK results in effective inhibition of replication of influenza virus [80,81,82]. JNK, another representative signaling molecule, promotes replication of viruses within host cells infected with influenza virus [83,84,85]. Many studies on attempts to treat influenza virus infection using inhibitors of p38 MAPK and JNK have been reported thus far [82]. However, effective suppression of activation of p38 MAPK and JNK by combination zanamivir/alloferon was confirmed in this study (Figure 3 and Figure 7). Therefore, the replication of influenza A virus is controlled by combination alloferon/zanamivir through inhibition of the activity of p38 MAPK and JNK. This could be the basis for the development of effective therapeutic agents through combinations of other drugs with alloferon or zanamivir for treatment of infection with influenza A virus.

Considering the findings of this study, the data presented herein demonstrate that combination alloferon/zanamivir can (1) inhibit virus-mediated activation of p38 MAPK and JNK; (2) inhibit production of inflammatory cytokines; and (3) inhibit infiltration of inflammatory cells into lung tissue; therefore, it can be regarded as an effective treatment for influenza A virus infection. In addition, our data provide a rationale for the development of therapeutic agents based on the combination of alloferon and zanamivir to control of the development and progression of chronic inflammatory lung diseases, as well as infection with influenza A virus.

## 4. Materials and Methods

### 4.1. Cells and Preparation of Virus Stocks

The human lung epithelial cell line A549 was maintained in RPMI 1640 (Welgene, Daegu, Korea), and MDCK and Calu-3 were maintained in Dulbecco’s modified Eagle’s medium (DMEM) (Welgene). Both media contained 10% heat-inactivated fetal bovine serum (Gibco, Queensland, Australia) and antibiotics (100 U/mL penicillin and 100 µg/mL streptomycin; Welgene). Cells were cultured at 37 °C in a humidified atmosphere containing 5% CO_2_. Isolation and passage of influenza A viruses was performed using MDCK cells. The influenza A/Puerto Rico/8/1934 was generously provided by Nam Hyuk Cho (Department of Microbiology, Seoul National University College of Medicine, Seoul, Korea). All animal experiments using influenza A virus were performed in an Animal Biosafety Level 2 facility at Seoul National University.

### 4.2. Propagation of Influenza A Virus (H1N1)

Influenza A/H1N1 viruses were propagated in MDCK cells cultured in DMEM (Welgene), supplemented with tosyl phenylalanyl chloromethyl ketone (TPCK)-treated trypsin (2 µg/mL; Sigma-Aldrich, St. Louis, MO, USA), harvested at 72 h post-infection, and titer testing was performed using a plaque assay based on MDCK [86].

### 4.3. Plaque Forming Unit Assay

MDCK (5.0 × 10^5^/well) and A549 (4.0 × 10^5^/well) cells were seeded onto six well plates at 37 °C in a 5% CO_2_ atmosphere. After overnight culture, washing of the confluent monolayer of MDCK cells was performed twice using 1× PBS buffer, followed by *treatment with a 10-fold serial dilution of* H1N1 influenza virus in serum-free medium containing only 1% penicillin streptomycin (Welgene). The plates were incubated for 1 h at 37 °C with constant agitation to induce viral infection. Washing of the monolayers with 1 × PBS buffer was performed for removal of virus, followed by application of 2 mL of agar overlay medium composed of 2× DMEM (Welgene), 2 µg/mL TPCK-treated trypsin, and *1.6%* agar (Lonza Bioscience, Morrisville, NC, USA). The incubation of the plates was then performed for three days at 37 °C under 5% CO_2_ and 95% relative humidity. The agar-covered monolayers were fixed with 4% para-formalin (Daejung, Siheung-si, Korea). Following removal of agar, staining of the fixed cells was performed using 0.5% crystal violet solution (Sigma-Aldrich, St. Loise, MO, USA) and plaques were counted.

### 4.4. Titration in a HA (Hemagglutination) Assay

Measurement of virus propagation was performed using an HA assay in virus-containing culture medium. Serial dilution of virus supernatants in saline in 96-well U-bottom plates was performed. Each well was then filled with 50 µL of a 0.5% suspension of human red blood cells (RBC). The plates were then allowed to sit at room temperature for 30 min. Following completion of the incubation period, an analysis of the assay was performed to determine RBC agglutination in each well. Across a row, agglutinated wells containing high concentrations of virus and exhibiting a diffuse reddish appearance progressed to wells with low concentrations of virus, which contained a dark red pellet. The results from the low concentration wells were nearly identical to those observed in the no-virus negative control wells. RBCs settled to the lowest point of the U-bottom well, resulting in a button-like appearance. Therefore, based on the experiments above, we measured the effective viral titer of HA (1:32 diluted in saline) before intranasally infecting mice (Supplementary Figure S5).

### 4.5. Animal Experiments

C57BL/6 wild-type mice were housed under pathogen-free conditions at the Seoul National University College of Medicine. The experiments were conducted using eight-week-old males. All experiments using animals were reviewed and approved by the Institutional Animal Care and Use Committee of Seoul National University (IACUC: SNU-200319-2). In this experiment, total five group, 1. Control (No infection) group, 2. H1N1 infection group, 3. H1N1 + Alloferon 0.5 µg/mL group, 4. H1N1 + Zanamivir 35 µg/mL, 5. H1N1 + Alloferon 0.5 µg/mL + Zanamivir 35 µg/mL group, (n = 30/group) for in vivo experiments. Therefore, results represent more than three independent experiments. Intranasal administration of influenza A/H1N1 virus in mice was performed under isoflurane anesthesia. Drugs of alloferon (0.5 µg/mL) and zanamivir (35 µg/mL) were administered daily following intranasal inoculation of H1N1. Monitoring of symptoms and mortality was performed daily for eight days. Over the course of eight days, measurements of body weight, clinical signs: 1. Changes in activity/mobility (Decreased mobility may be localized to area of pain) 2. Behavior changes during handling (placid) 3. Separation from group (Mice are social and normally rest in close proximity to one another)], and survival were performed daily. Mice with weight loss exceeding 30%, or mice that were clearly terminal were euthanized in accordance with guidelines established by the animal ethics committee.

### 4.6. Serum and BALF Collection

Blood samples were collected from the retro-orbital plexus of each animal using heparinized capillaries and immediately centrifuged at 3000 rpm for 10 min at 4 °C to obtain serum. For BALF samples, PBS containing 1% bovine serum albumin and 0.1% NaN_3_ (blocking solution) was used to lavage the trachea and lungs. After centrifuging the lavage fluid for 10 min at 300× *g,* the supernatant was stored at –80 °C [87]. The levels of TNF-α, IL-6, IL-1β, IFN-λ, MIP-1α, and IL-10 in serum and BALF were measured using ELISA kits (BioLegend, San Diego, CA, USA).

### 4.7. Western Blot Analysis

Western blotting was performed in order to evaluate the expression of proteins involved in signaling pathways. Protein lysate was obtained from lung tissue of H1N1 infected mice and MDCK and A549 cells treated with alloferon and zanamivir for 12 and 24 h after infection with H1N1 using lysis buffer containing 50 mM Tris-HCL (pH 7.4), 1% NP-40, 0.25% sodium deoxycholate, 150 mM NaCl, 1 mM EDTA, and protease inhibitor cocktails (Sigma-Aldrich). The BCA assay was performed for quantification of the protein concentration by BCA kit (Sigma-Aldrich). Then, measurement of the relative absorbance at 562 nm was performed using a SpectraMax iD3 microplate reader (Molecular Devices, San Jose, CA, USA). The proteins (20 µg/sample) were resolved in a 10% polyacrylamide-SDS gel at 100 V for 2 h, followed by transfer to nitrocellulose membranes. Blocking was performed for 1 h at room temperature with 5% nonfat milk in PBS. Incubation of the blocked membrane was performed at 4 °C overnight with antibodies specific for p-p38 (1:1000; Cell Signaling; Boston, MA, USA), p-c-Jun (1:1000; Cell Signaling), p38 (1:1000; Cell Signaling), c-Jun (1:1000; Cell Signaling) or anti-β-actin Ab (1:5000; Santa Cruz Biotechnology; Santa Cruz, CA, USA). The membrane was washed three times (5 min each) with 0.1% PBST, the membrane was followed by incubation for 1 h at room temperature with horseradish peroxidase (HRP)-conjugated anti-rabbit IgG antibodies (1:5000; Cell Signaling Technology) for detection of p-p38, p-c-Jun, p38, and c-Jun, or with HRP-conjugated anti-mouse IgG antibody (1:10,000; Cell Signaling) for detection of β-actin. An electrochemical luminescence detection system (Thermo Scientific, Wilmington, DE, USA) was used for the visualization of the immunoreactive proteins after washing three times (5 min each). Analysis of the density of the bands was performed using Image J software (NIH), and the phospho form was normalized to the total form.

### 4.8. Quantification of Virus Replication by Real-Time RT-PCR Analysis

Extraction of total RNA from A549 and MDCK cells at 12 and 24 h post-infection and lung specimens of H1N1-infected mice was performed using Trizol Reagent (Invitrogen, Grand Island, NY, USA). Reverse transcription of RNA samples into cDNAs was performed using oligo (dT) primers and AMV reverse transcriptase (iNtRON; Daejeon, Korea). Briefly, 1 µg of total RNA was combined with 5 mM MgCl_2_, 1 mM of each dNTP, 1 µL of RT buffer, 1 U/mL RNasin, 15 U/mL reverse transcriptase, and DEPC treated water up to a total volume of 20 µL. The samples were incubated for 10 min at room temperature, followed by incubation for 1 h at 42 °C, 5 min at 72 °C, followed by another 5 min on ice. Following addition of 180 μL of DEPC treated water, samples were stored at −70 °C. Each reaction was run in a quadrant for each sample, and an analysis of the results was performed using the Rotor-Gene SYBR Green PCR kit (Qiagen, Hilden, Germany) and Rotor-Gene Q 2plex-real-time RT PCR instrument (Qiagen). The reaction mixture (20 µL) consisted of 2 µL of a cDNA template and 1.5 µL each of primers. Amplification was performed using SYBR Green master mix (Qiagen). Each cycle of PCR was monitored by measuring the fluorescence produced by binding of SYBR Green dye to dsDNA. The following primers were used: H1; Forward 5′-AGCAAAAGCAGGGGAAAATAAAA-3′; Reverse 5′-CACGAGGACTTCTTTCCCTTTATCAT-3′; and GAPDH; Forward 5′-GGTGGTCCAGGGTTTCTTA-3′; Reverse 5′-GTTGTCTCCTGCGACTTCA-3′. Calculation of differences in the amount of H1N1 was performed using the using the ∆∆CT method [88].

### 4.9. Enzyme-Linked Immunosorbent Assay (ELISA)

A549 (2 × 10^5^/well) and MDCK (1 × 10^5^/well) cells were seeded into six well plates overnight, followed by treatment with alloferon (0.5 µg/mL) and/or zanamivir (35 µg/mL) for 48 and 72 h after infection with H1N1 (MOI 0.01). Mouse lung tissues, BALF, and serum were stored at −80 °C. MIP-1α and IL-6 levels were determined using IL-6 and MIP-1α BioLegend’s ELISA MAX™ Standard Set Kits according to manufacturer’s protocol based on Avidin-Biotin reaction (BioLegend; San Diego, CA, USA). Measurement of the relative absorbance at 450 nm was performed using a SpectraMax iD3 microplate reader (Molecular Devices).

### 4.10. Histopathology

Lung tissues were harvested and fixed overnight in 4% Paraformaldehyde (PFA) at 4 °C. Fixed tissues were embedded in paraffin prior to cutting of sections with 4 µm thickness. Following deparaffinization and hydration, the sections were stained with hematoxylin and eosin, followed by viewing under a light microscope. Masson’s Trichrome staining was performed using a VitroView^TM^ Masson’s Trichrome Stain Kit (VitroView^TM^, Hanam, Korea). Briefly, Weigert’s iron hematoxylin, Biebrich Scarlet-Acid Fuchsin, and Aniline Blue Stain solutions were applied to deparaffinized sections pretreated with Bouin’s fluid at 56 °C for 1 h. Finally, removal of non specific staining was performed using 1% acetic acid solution. The nuclei were then stained black, the cytoplasm and muscle tissue were stained red, and collagen was stained blue. Alveoli in the lungs did not show an even distribution. Although scanning of whole-mount lung sections was not performed for measurement of collagen in this study, Trichrome staining of lung tissue enabled an estimation of differences in the amount of collagen. All analyses were performed using Celleste (Thermo Scientific).

### 4.11. Statistical Analysis

Data are presented as the mean ± SD. The comparison of data between groups was performed using unpaired t-tests. Statistical analysis was performed using GraphPad Software Prism version 6.01 (GraphPad Software, La Jolla, CA, USA).

## 5. Conclusions

The combination of alloferon and zanamivir can be regarded as an effective treatment for influenza A virus infection by (1) inhibiting the activity of p38 MAPK and JNK; (2) inhibiting production of inflammatory cytokine in the lungs; and (3) inhibiting the infiltration of inflammatory cells into lung tissue. In addition, there is the potential for the use of alloferon/zanamivir in the development of other therapeutic agents capable of regulating the progression of chronic inflammatory lung diseases and influenza A/H1N1 infection.

## Data Availability

All datasets generated for this study are included in the article or in the Supplementary File.

## Supplementary Materials

The following supporting information can be downloaded at: https://www.mdpi.com/article/10.3390/ijms24010678/s1.

## References

[1] Kaur N., Singh R., Dar Z., Bijarnia R.K., Dhingra N., Kaur T. (2021). Genetic comparison among various coronavirus strains for the identification of potential vaccine targets of SARS-CoV2. Infect. Genet. Evol..

[2] Alluwaimi A.M., Alshubaith I.H., Al-Ali A.M., Abohelaika S. (2020). The Coronaviruses of Animals and Birds: Their Zoonosis, Vaccines, and Models for SARS-CoV and SARS-CoV2. Front. Vet. Sci..

[3] Torresi J., Edeling M.A., Nolan T., Godfrey D.I. (2022). A complementary union of SARS-CoV2 natural and vaccine induced immune responses. Front. Immunol..

[4] Yu Z., Ellahi R., Nutini A., Sohail A., Sait S.M. (2021). Modeling and simulations of CoViD-19 molecular mechanism induced by cytokines storm during SARS-CoV2 infection. J. Mol. Liq..

[5] Vanderbeke L., Spriet I., Breynaert C., Rijnders B.J.A., Verweij P.E., Wauters J. (2018). Invasive pulmonary aspergillosis complicating severe influenza: Epidemiology, diagnosis and treatment. Curr. Opin. Infect. Dis..

[6] Kalil A.C., Thomas P.G. (2019). Influenza virus-related critical illness: Pathophysiology and epidemiology. Crit. Care.

[7] Yue H., Zhang M., Xing L., Wang K., Rao X., Liu H., Tian J., Zhou P., Deng Y., Shang J. (2020). The epidemiology and clinical characteristics of co-infection of SARS-CoV-2 and influenza viruses in patients during COVID-19 outbreak. J. Med. Virol..

[8] Hause B.M., Collin E.A., Liu R., Huang B., Sheng Z., Lu W., Wang D., Nelson E.A., Li F. (2014). Characterization of a Novel Influenza Virus in Cattle and Swine: Proposal for a New Genus in the *Orthomyxoviridae* Family. Mbio.

[9] Cheung T.K.W., Poon L.L.M. (2007). Biology of Influenza A Virus. Ann. N. Y. Acad. Sci..

[10] Asadi S., ben Hnia N.G., Barre R.S., Wexler A.S., Ristenpart W.D., Bouvier N.M. (2020). Influenza A virus is transmissible via aerosolized fomites. Nat. Commun..

[11] Traxler S., Bischoff A.-C., Saß R., Trefz P., Gierschner P., Brock B., Schwaiger T., Karte C., Blohm U., Schröder C. (2018). VOC breath profile in spontaneously breathing awake swine during Influenza A infection. Sci. Rep..

[12] Medina R.A., Garcia-Sastre A. (2011). Influenza A viruses: New research developments. Nat. Rev. Genet..

[13] Bailey E.S., Fieldhouse J.K., Choi J.Y., Gray G.C. (2018). A Mini Review of the Zoonotic Threat Potential of Influenza Viruses, Coronaviruses, Adenoviruses, and Enteroviruses. Front. Public Health.

[14] Baigent S.J., McCauley J.W. (2003). Influenza type A in humans, mammals and birds: Determinants of virus virulence, host-range and interspecies transmission. Bioessays.

[15] Kang H.-J., Chu K.-B., Yoon K.-W., Eom G.-D., Mao J., Kim M.-J., Lee S.-H., Moon E.-K., Quan F.-S. (2021). Neuraminidase in Virus-like Particles Contributes to the Protection against High Dose of Avian Influenza Virus Challenge Infection. Pathogens.

[16] Sempere Borau M., Stertz S. (2021). Entry of influenza A virus into host cells-recent progress and remaining challenges. Curr. Opin. Virol..

[17] Chivukula S., Plitnik T., Tibbitts T., Karve S., Dias A., Zhang D., Goldman R., Gopani H., Khanmohammed A., Sarode A. (2021). Development of multivalent mRNA vaccine candidates for seasonal or pandemic influenza. NPJ Vaccines.

[18] Du W., de Vries E., van Kuppeveld F.J.M., Matrosovich M., de Haan C.A.M. (2021). Second sialic acid-binding site of influenza A virus neuraminidase: Binding receptors for efficient release. FEBS J..

[19] Sugaya N., Tamura D., Yamazaki M., Ichikawa M., Kawakami C., Kawaoka Y., Mitamura K. (2008). Comparison of the Clinical Effectiveness of Oseltamivir and Zanamivir against Influenza Virus Infection in Children. Clin. Infect. Dis..

[20] Tappenden P., Jackson R., Cooper K., Rees A., Simpson E., Read R., Nicholson K. (2009). Amantadine, oseltamivir and zanamivir for the prophylaxis of influenza (including a review of existing guidance no. 67): A systematic review and economic evaluation. Health Technol. Assess..

[21] Ryu M.J., Anikin V., Hong S.H., Jeon H., Yu Y.G., Yu M.H., Chernysh S., Lee C. (2008). Activation of NF-kappaB by alloferon through down-regulation of antioxidant proteins and IkappaBalpha. Mol. Cell. Biochem..

[22] Matusiak A., Kuczer M., Czarniewska E., Rosiński G., Kowalik-Jankowska T. (2014). Copper(II) complexes of alloferon 1 with point mutations (H1A) and (H9A) stability structure and biological activity. J. Inorg. Biochem..

[23] Kim Y., Lee S.K., Bae S., Kim H., Park Y., Chu N.K., Kim S.G., Kim H.-R., Hwang Y.-I., Kang J.S. (2013). The anti-inflammatory effect of alloferon on UVB-induced skin inflammation through the down-regulation of pro-inflammatory cytokines. Immunol. Lett..

[24] Lee N., Bae S., Kim H., Kong J.M., Kim H.R., Cho B.J., Kim S.J., Seok S.H., Hwang Y., Kim S. (2011). Inhibition of lytic reactivation of Kaposi’s sarcoma-associated herpesvirus by alloferon. Antivir. Ther..

[25] Jeon J., Kim Y., Kim H., Kang J.S., Lee W.J. (2015). Anti-inflammatory Effect of Alloferon on Ovalbumin-induced Asthma. Immune Netw..

[26] Tyno Y.Y., Jarigina E.I., Ustinova V.A., Vidrashko M.T., Morosova G.V., Bakaeva E.V. (2018). Antiviral Activity of Allokin-Alpha against Herpesvirus in Birds. Russ. Agric. Sci..

[27] Bae S., Oh K., Kim H., Kim Y., Kim H.-R., Hwang Y.-I., Lee D.-S., Kang J.S., Lee W.J. (2013). The effect of alloferon on the enhancement of NK cell cytotoxicity against cancer via the up-regulation of perforin/granzyme B secretion. Immunobiology.

[28] Chernysh S., Kim S.I., Bekker G., Pleskach V.A., Filatova N.A., Anikin V.B., Platonov V.G., Bulet P. (2002). Antiviral and antitumor peptides from insects. Proc. Natl. Acad. Sci. USA.

[29] Chernysh S.I., Gordja N.A. (2011). The immune system of maggots of the blow fly (Calliphora vicina) as a source of medicinal drugs. J. Evol. Biochem. Physiol..

[30] Mancilla-Galindo J., García-Méndez J., Márquez-Sánchez J., Reyes-Casarrubias R.E., Aguirre-Aguilar E., Rocha-González H.I., Kammar-García A. (2021). All-cause mortality among patients treated with repurposed antivirals and antibiotics for COVID-19 in Mexico City: A real-world observational study. EXCLI J..

[31] Jackson R.J., Cooper K.L., Tappenden P., Rees A., Simpson E.L., Read R.C., Nicholson K.G. (2011). Oseltamivir, zanamivir and amantadine in the prevention of influenza: A systematic review. J. Infect..

[32] Clark N.M., Lynch J.P. (2011). Influenza: Epidemiology, clinical features, therapy, and prevention. Semin. Respir. Crit. Care Med..

[33] Dabbu Kumar J., Jian L., Rong H., Hua Z. (2018). Emerging and reemerging human viral diseases. Ann. Microbiol. Res..

[34] Alkan Ozdemir S., Soysal B., Calkavur S., Gokmen Yildirim T., Kiymet E., Kalkanli O., Çolak R., Devrim İ. (2022). Is respiratory syncytial virus infection more dangerous than COVID 19 in the neonatal period?. J. Matern. Fetal Neonatal Med..

[35] Peltola V., Shih S.R., To K.K. (2020). Editorial: Respiratory Virus Infection: Recent Advances. Front. Med. Lausanne.

[36] Shim S., Chan M., Owens L., Jaffe A., Prentice B., Homaira N. (2021). Rate of use and effectiveness of oseltamivir in the treatment of influenza illness in high-risk populations: A systematic review and meta-analysis. Health Sci. Rep..

[37] Mott J.A., Fry A.M., Kondor R., Wentworth D.E., Olsen S.J. (2021). Re-emergence of influenza virus circulation during 2020 in parts of tropical Asia: Implications for other countries. Influ. Other Respir. Viruses.

[38] Holmes E.C., Hurt A.C., Dobbie Z., Clinch B., Oxford J.S., Piedra P.A. (2021). Understanding the Impact of Resistance to Influenza Antivirals. Clin. Microbiol. Rev..

[39] Ison M.G., Hayden F.G., Hay A.J., Gubareva L.V., Govorkova E.A., Takashita E., McKimm-Breschkin J.L. (2021). Influenza polymerase inhibitor resistance: Assessment of the current state of the art—A report of the isirv Antiviral group. Antivir. Res..

[40] Underwood M., Horton J., Nangle K., Hopking J., Smith K., Aboud M., Wynne B., Sievers J., Stewart E.L., Wang R. (2022). Integrase Inhibitor Resistance Mechanisms and Structural Characteristics in Antiretroviral Therapy-Experienced, Integrase Inhibitor-Naive Adults with HIV-1 Infection Treated with Dolutegravir plus Two Nucleoside Reverse Transcriptase Inhibitors in the DAWNING Study. Antimicrob. Agents Chemother..

[41] Pushko P., Tumpey T.M., Bu F., Knell J., Robinson R., Smith G. (2005). Influenza virus-like particles comprised of the HA, NA, and M1 proteins of H9N2 influenza virus induce protective immune responses in BALB/c mice. Vaccine.

[42] Leser G.P., Lamb R.A. (2005). Influenza virus assembly and budding in raft-derived microdomains: A quantitative analysis of the surface distribution of HA, NA and M2 proteins. Virology.

[43] Medeiros J.J.S., Costa T.M., Carmo M.P., Nascimento D.D., Lauro E.N.C., Oliveira C.A., Duque M.D., Prado L.D. (2022). Efficient drug development of oseltamivir capsules based on process control, bioequivalence and PBPK modeling. Drug Dev. Ind. Pharm..

[44] Zhang W., Xu H., Guan S., Wang C., Dong G. (2022). Frequency and distribution of H1N1 influenza A viruses with oseltamivir-resistant mutations worldwide before and after the 2009 pandemic. J. Med. Virol..

[45] Jeyaram R.A., Anu Radha C. (2021). N1 neuraminidase of H5N1 avian influenza A virus complexed with sialic acid and zanamivir—A study by molecular docking and molecular dynamics simulation. J. Biomol. Struct. Dyn..

[46] Duan C., Liu W., Bi S., Chen L., Pan J., Zhou T., Lin K., Zhou W. (2021). Synthesis and characterization of 4(*R*)-epimer impurities of zanamivir and laninamivir octanoate. J. Heterocycl. Chem..

[47] Liu X., Luo W., Zhang B., Lee Y.G., Shahriar I., Srinivasarao M., Low P.S. (2021). Design of Neuraminidase-Targeted Imaging and Therapeutic Agents for the Diagnosis and Treatment of Influenza Virus Infections. Bioconjugate Chem..

[48] Gubareva L.V., Kaiser L., Hayden F.G. (2000). Influenza virus neuraminidase inhibitors. Lancet.

[49] McAuley J.L., Gilbertson B., Trifkovic S., Brown L.E., McKimm-Breschkin J.L. (2019). Influenza Virus Neuraminidase Structure and Functions. Front. Microbiol..

[50] Chiaretti A., Pulitano S., Barone G., Ferrara P., Romano V., Capozzi D., Riccardi R. (2013). IL-1 beta and IL-6 upregulation in children with H1N1 influenza virus infection. Mediat. Inflamm..

[51] Dienz O., Rud J.G., Eaton S.M., A Lanthier P., Burg E., Drew A., Bunn J., Suratt B.T., Haynes L., Rincon M. (2012). Essential role of IL-6 in protection against H1N1 influenza virus by promoting neutrophil survival in the lung. Mucosal Immunol..

[52] Trammell R.A., Liberati T.A., Toth L.A. (2012). Host genetic background and the innate inflammatory response of lung to influenza virus. Microbes Infect..

[53] Forbester J.L., Humphreys I.R. (2020). Genetic influences on viral-induced cytokine responses in the lung. Mucosal Immunol..

[54] Jo H., Lee D., Go C., Jang Y., Bae S., Agura T., Hong J., Kang D., Kim Y., Kang J.S. (2022). Alloferon Affects the Chemosensitivity of Pancreatic Cancer by Regulating the Expression of SLC6A14. Biomedicines.

[55] Kim H., Im J.P., Kim J.S., Kang J.S., Lee W.J. (2015). Alloferon Alleviates Dextran Sulfate Sodium-induced Colitis. Immune Netw..

[56] Ishiguro N., Koseki N., Kaiho M., Ariga T., Kikuta H., Oba K., Togashi T., Morita K., Inagawa A., Okamura A. (2018). Clinical effectiveness of four neuraminidase inhibitors (oseltamivir, zanamivir, laninamivir, and peramivir) for children with influenza A and B in the 2014–2015 to 2016–2017 influenza seasons in Japan. J. Infect. Chemother..

[57] Yin H., Jiang N., Shi W., Chi X., Liu S., Chen J.-L., Wang S. (2021). Development and Effects of Influenza Antiviral Drugs. Molecules.

[58] Sette D.E.S.P.H., Costa M.J.F., Araujo F.A.C., Alencar E.N., Amaral-Machado L. (2021). Two phytocompounds from Schinopsis brasiliensis show promising antiviral activity with multiples targets in Influenza A virus. An. Acad. Bras. Cienc..

[59] Eryildiz B., Gul B.Y., Koyuncu I. (2022). A sustainable approach for the removal methods and analytical determination methods of antiviral drugs from water/wastewater: A review. J. Water Process Eng..

[60] Hurt A.C., Holien J.K., Parker M., Kelso A., Barr I.G. (2009). Zanamivir-Resistant Influenza Viruses with a Novel Neuraminidase Mutation. J. Virol..

[61] Trebbien R., Pedersen S.S., Vorborg K., Franck K.T., Fischer T.K. (2017). Development of oseltamivir and zanamivir resistance in influenza A(H1N1)pdm09 virus, Denmark, 2014. Eurosurveillance.

[62] Qin H.-J., Li S., Zhu Y.-B., Bao Y.-B., Tang Q., Liu W.-B., Zhong M., Zhao Y., Yang Y. (2022). Oseltamivir modified bovine serum albumin inhibits neuraminidase activity and accumulates virion particles to disturb influenza virus replication. Carbohydr. Res..

[63] Hsiao T.-C., Cheng P.-C., Chi K.H., Wang H.-Y., Pan S.-Y., Kao C., Lee Y.-L., Kuo H.-P., Chung K.F., Chuang H.-C. (2021). Interactions of chemical components in ambient PM2.5 with influenza viruses. J. Hazard. Mater..

[64] Mahal A., Duan M., Zinad D.S., Mohapatra R.K., Obaidullah A.J., Wei X., Pradhan M.K., Das D., Kandi V., Zinad H.S. (2021). Recent progress in chemical approaches for the development of novel neuraminidase inhibitors. RSC Adv..

[65] Dohna-Schwake C., Schweiger B., Felderhoff-Müser U., Fiedler M., Kaiser G.M., Paul A., Gerner P., Lainka E., Hoyer P.F. (2010). Severe H1N1 Infection in a Pediatric Liver Transplant Recipient Treated With Intravenous Zanamivir: Efficiency and Complications. Transplantation.

[66] Kaminski M.M., Ohnemus A., Staeheli P., Rubbenstroth D. (2013). Pandemic 2009 H1N1 Influenza A Virus Carrying a Q136K Mutation in the Neuraminidase Gene Is Resistant to Zanamivir but Exhibits Reduced Fitness in the Guinea Pig Transmission Model. J. Virol..

[67] Aleebrahim-Dehkordi E., Molavi B., Mokhtari M., Deravi N., Fathi M., Fazel T., Mohebalizadeh M., Koochaki P., Shobeiri P., Hasanpour-Dehkordi A. (2022). T helper type (Th1/Th2) responses to SARS-CoV-2 and influenza A (H1N1) virus: From cytokines produced to immune responses. Transpl. Immunol..

[68] Wohlleben G., Müller J., Tatsch U., Hambrecht C., Herz U., Renz H., Schmitt E., Moll H., Erb K.J. (2003). Influenza A Virus Infection Inhibits the Efficient Recruitment of Th2 Cells into the Airways and the Development of Airway Eosinophilia. J. Immunol..

[69] Morris G., Bortolasci C.C., Puri B.K., Marx W., O’Neil A., Athan E., Walder K., Berk M., Olive L., Carvalho A.F. (2021). The cytokine storms of COVID-19, H1N1 influenza, CRS and MAS compared. Can one sized treatment fit all?. Cytokine.

[70] Wu Y., Li Y.-Y., Matsushima K., Baba T., Mukaida N. (2008). CCL3-CCR5 axis regulates intratumoral accumulation of leukocytes and fibroblasts and promotes angiogenesis in murine lung metastasis process. J. Immunol..

[71] Struyf S., Schutyser E., Gouwy M., Gijsbers K., Proost P., Benoit Y., Opdenakker G., Van Damme J., Laureys G. (2003). PARC/CCL18 Is a Plasma CC Chemokine with Increased Levels in Childhood Acute Lymphoblastic Leukemia. Am. J. Pathol..

[72] Menten P., Saccani A., Dillen C., Wuyts A., Struyf S., Proost P., Mantovani A., Wang J.M., van Damme J. (2002). Role of the autocrine chemokines MIP-1α and MIP-1β in the metastatic behavior of murine T cell lymphoma. J. Leukoc. Biol..

[73] Patricia Menten A.W., van Damme J. (2002). Macrophage inflammatory protein-1. Cytokine Growth Factor Rev..

[74] Bhavsar I., Miller C.S., Al-Sabbagh M. (2015). Macrophage inflammatory protein-1 alpha (MIP-1 alpha)/CCL3: As a biomarker. Gen. Methods Biomark. Res. Appl..

[75] Rudnicka E., Suchta K., Grymowicz M., Calik-Ksepka A., Smolarczyk K., Duszewska A.M., Smolarczyk R., Meczekalski B. (2021). Chronic Low Grade Inflammation in Pathogenesis of PCOS. Int. J. Mol. Sci..

[76] Garofalo R., Kimpen J.L., Welliver R.C., Ogra P.L. (1992). Eosinophil degranulation in the respiratory tract during naturally acquired respiratory syncytial virus infection. J. Pediatr..

[77] Jorquera P.A., Tripp R.A. (2016). Synthetic Biodegradable Microparticle and Nanoparticle Vaccines against the Respiratory Syncytial Virus. Vaccines.

[78] Cook D.N. (1996). The role of MIP-1α in Inflammation and hematopoiesis. J. Leukoc. Biol..

[79] Goodman R.B., Strieter R.M., Martin D.P., Steinberg K.P., A Milberg J., Maunder R.J., Kunkel S.L., Walz A., Hudson L.D., Martin T.R. (1996). Inflammatory cytokines in patients with persistence of the acute respiratory distress syndrome. Am. J. Respir. Crit. Care Med..

[80] Börgeling Y., Schmolke M., Viemann D., Nordhoff C., Roth J., Ludwig S. (2014). Inhibition of p38 Mitogen-activated Protein Kinase Impairs Influenza Virus-induced Primary and Secondary Host Gene Responses and Protects Mice from Lethal H5N1 Infection. J. Biol. Chem..

[81] Choi M.-S., Heo J., Yi C.-M., Ban J., Lee N.-J., Lee N.-R., Kim S.W., Kim N.-J., Inn K.-S. (2016). A novel p38 mitogen activated protein kinase (MAPK) specific inhibitor suppresses respiratory syncytial virus and influenza A virus replication by inhibiting virus-induced p38 MAPK activation. Biochem. Biophys. Res. Commun..

[82] Dai J.-P., Wang Q.-W., Su Y., Gu L.-M., Deng H.-X., Chen X.-X., Li W.-Z., Li K.-S. (2018). Oxymatrine Inhibits Influenza A Virus Replication and Inflammation via TLR4, p38 MAPK and NF-κB Pathways. Int. J. Mol. Sci..

[83] Zhang J., Ruan T., Sheng T., Wang J., Sun J., Wang J., Prinz R.A., Peng D., Liu X., Xu X. (2018). Role of c-Jun terminal kinase (JNK) activation in influenza A virus-induced autophagy and replication. Virology.

[84] Nacken W., Wixler V., Ehrhardt C., Ludwig S. (2017). Influenza A virus NS1 protein-induced JNK activation and apoptosis are not functionally linked. Cell. Microbiol..

[85] Tang Y., Yang G., Li Y., Wang M., Li G., Hu Y. (2021). Protective effects of SP600125 on mice infected with H1N1 influenza A virus. Arch. Virol..

[86] Gambaryan A.S., Karasin A.I., Tuzikov A.B., Chinarev A.A., Pazynina G.V., Bovin N.V., Matrosovich M., Olsen C.W., Klimov A.I. (2005). Receptor-binding properties of swine influenza viruses isolated and propagated in MDCK cells. Virus Res..

[87] Daubeuf F., Frossard N. (2012). Performing Bronchoalveolar Lavage in the Mouse. Curr. Protoc. Mouse Biol..

[88] Liu S., Hou G., Zhuang Q., Shu Y., Chen J., Jiang W., Chen J., Li J. (2009). A SYBR Green I real-time RT-PCR assay for detection and differentiation of influenza A(H1N1) virus in swine populations. J. Virol. Methods.

