# Supplementary material for: Alloferon and Zanamivir Show Effective Antiviral Activity against Influenza A Virus (H1N1) Infection In Vitro and In Vivo
Source: Int J Mol Sci. 2022 Dec 30;24(1):678. doi: 10.3390/ijms24010678 (PMC9820929; doi:10.3390/ijms24010678)
Supplement: Supplementary file 1 [file ijms-24-00678-s001.zip › ijms-2052111-supplementary.pdf]

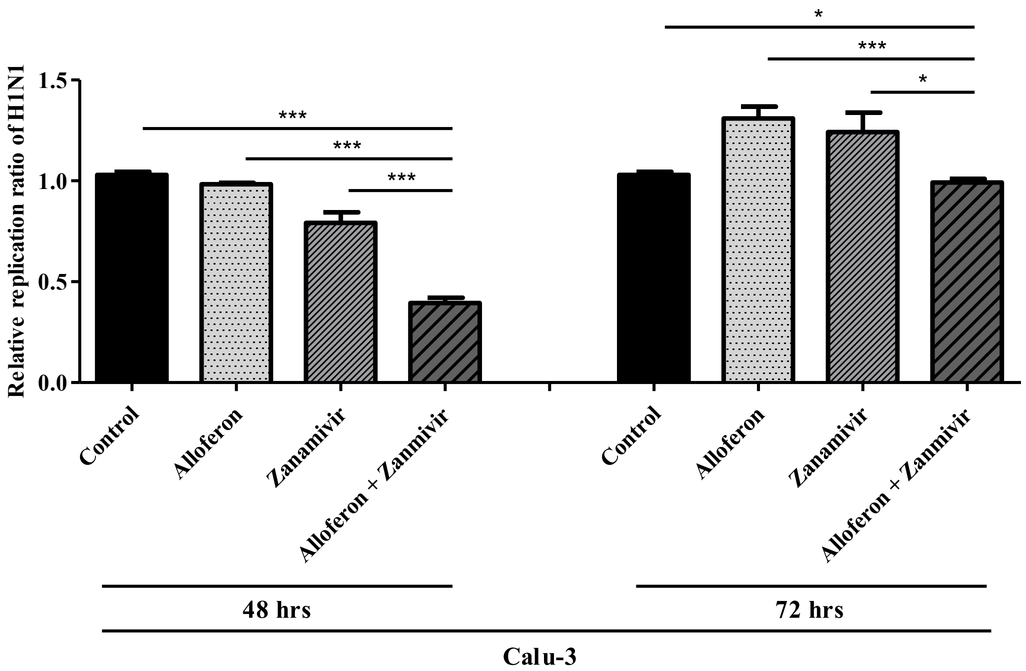

Figure S1

Inhibitory effects of alloferon and zanamivir on influenza A/H1N1 replication in Calu-3 cells.
Quantitative real-time PCR analysis of influenza virus levels (normalized to GAPDH). Cells were infected with influenza viruses at a multiplicity of infection of 0.01. After 1 h, viruses were removed and cells were treated for 48 and 72 h with serum-free medium containing alloferon (0.5 μg/mL) and/or zanamivir (35 μg/mL). At each time point, total RNA was extracted from cells and RT-PCR was performed using primers specific for H1N1. Results are representative of three independent experiments. *$p < 0.1$. **$p < 0.01$, ***$p < 0.001$.

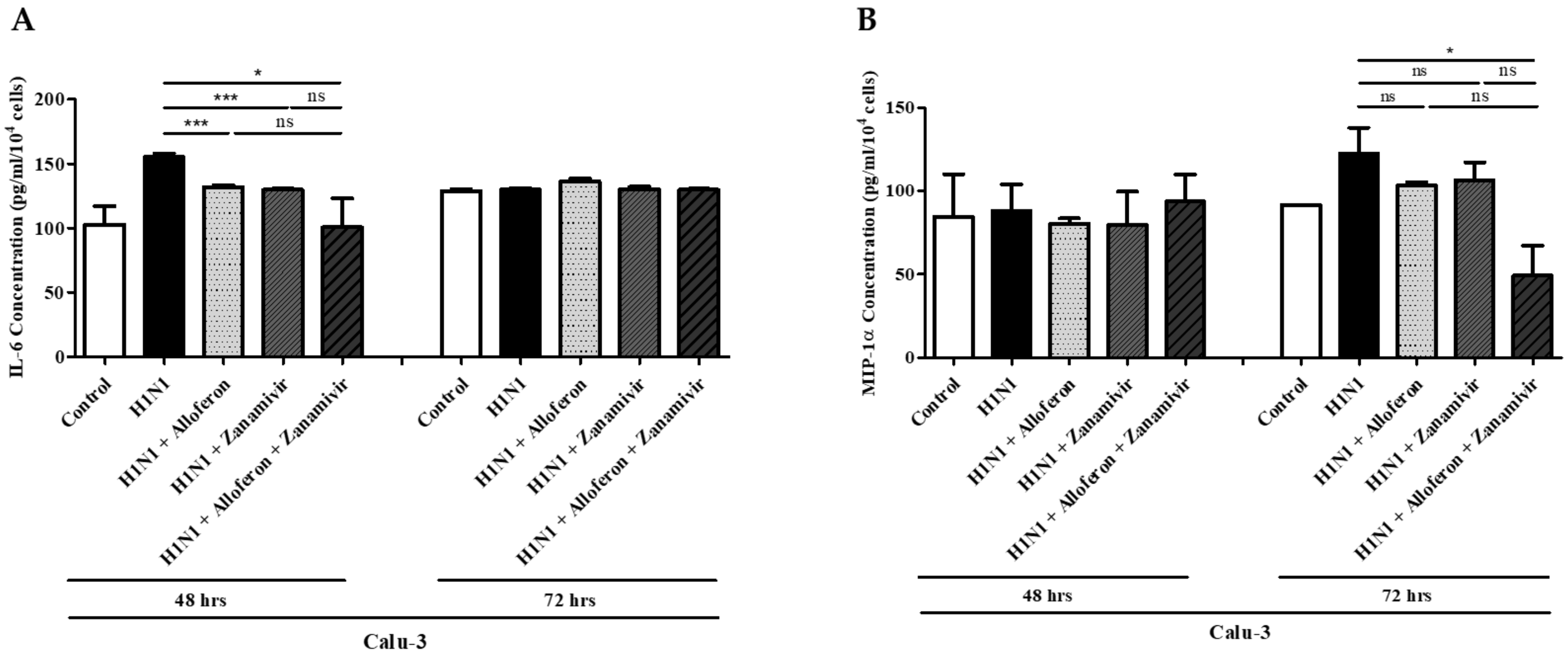

Figure S2

Pro-inflammatory cytokine production by Calu-3 cells infected with influenza A virus (H1N1).
IL-6 and MIP-1α production by Calu-3 cells infected with influenza A/H1N1 at an MOI of 0.01. At 1 h post-infection, viruses were removed from the cells and serum free medium containing alloferon (0.5 μg/mL) and/or zanamivir (35 μg/mL) was added. The culture supernatants were collected at 48 and 72 h and production of IL-6 and MIP-1α was measured in ELISAs. Results are representative of three independent experiments. *$p < 0.1$, **$p < 0.01$, ***$p < 0.001$. NS; No Significant.

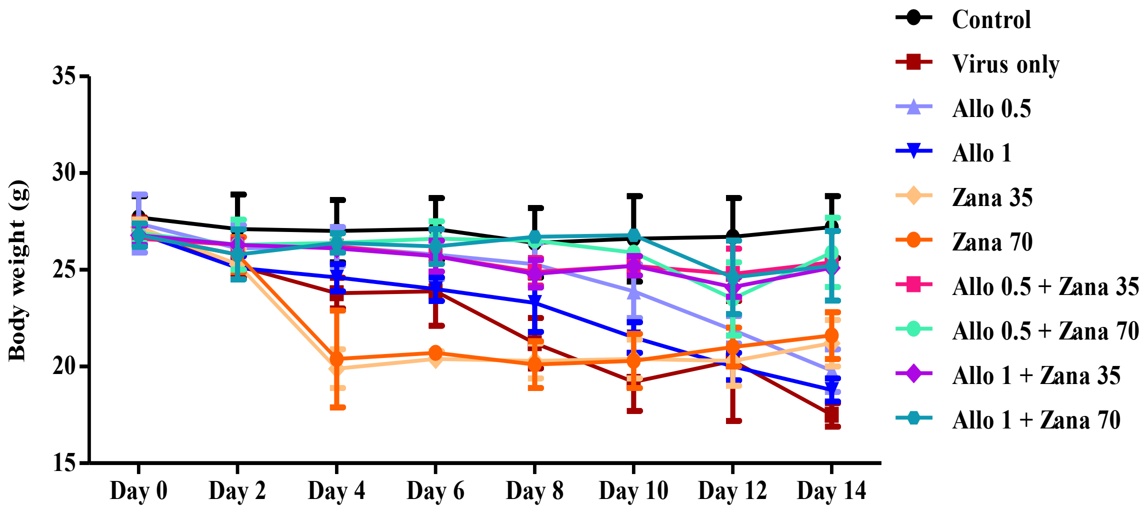

	Day 0	Day 2	Day 3	Day 4	Day 5	Day 6	Day 7	Day 8
**Control**	28.7	28.4	25.2	28.3	28.3	29.0	26.0	26.8
	27.9	27.8	28.4	25.2	24.8	24.7	29.0	28.9
	26.5	25.1	27.3	27.7	26.0	26.0	25.1	25.8
**AVERAGE**	**27.7**	**27.1**	**27.0**	**27.1**	**26.4**	**26.6**	**26.7**	**27.2**
**SD**	**1.1**	**1.8**	**1.6**	**1.6**	**1.8**	**2.2**	**2.0**	**1.6**
**Virus only**	27.7	24.7	23.8	22.1	20.5	18.1	23.7	17
	26.9	25.0	23.0	25.7	20.5	18.6	17.8	17.2
	26.9	25.9	24.6	23.8	22.7	20.9	19.4	18.2
**AVERAGE**	**27.2**	**25.2**	**23.8**	**23.9**	**21.2**	**19.2**	**20.3**	**17.5**
**SD**	**0.5**	**0.6**	**0.8**	**1.8**	**1.3**	**1.5**	**3.1**	**0.6**
**Allo (0.5)**	29.0	27.5	27.3	27.3	25.0	23.2	21.1	19.1
	27.1	25.5	25.9	25.1	24.4	23.0	22.3	19.3
	26.1	25.3	25.3	25.1	26.5	25.5	22.4	21.1
**AVERAGE**	**27.4**	**26.1**	**26.2**	**25.8**	**25.3**	**23.9**	**21.9**	**19.8**
**SD**	**1.5**	**1.2**	**1.0**	**1.3**	**1.1**	**1.4**	**0.7**	**1.1**
**Allo (1)**	26.5	24.9	25.4	23.5	24.9	22.4	19.4	18.2
	27.2	24.7	24.1	23.9	22.0	20.9	19.9	19.4
	27.1	25.8	24.2	24.6	23.0	21.1	20.8	18.8
**AVERAGE**	**26.9**	**25.1**	**24.6**	**24.0**	**23.3**	**21.5**	**20.0**	**18.8**
**SD**	**0.4**	**0.6**	**0.7**	**0.6**	**1.5**	**0.8**	**0.7**	**0.6**
**Zan(35)**	26.9	24.5	19.0	20.0	19.3	19.2	18.9	22.3
	27.6	25.2	21.0	20.7	20.9	20.9	20.5	21.4
	27.2	26.1	19.7	20.6	20.7	21.1	21.5	19.9
**AVERAGE**	**27.2**	**25.3**	**19.9**	**20.4**	**20.3**	**20.4**	**20.3**	**21.2**
**SD**	**0.4**	**0.8**	**1.0**	**0.4**	**0.9**	**1.0**	**1.3**	**1.2**
**Zan(70)**	26.8	26.6	20.9	20.5	21.1	19.0	20.5	20.9
	26.5	25.9	17.6	20.5	20.5	21.7	22.2	20.8
	27.1	24.8	22.6	21.1	18.7	20.1	20.3	23.0
**AVERAGE**	**26.8**	**25.8**	**20.4**	**20.7**	**20.1**	**20.3**	**21.0**	**21.6**
**SD**	**0.3**	**0.9**	**2.5**	**0.3**	**1.2**	**1.4**	**1.0**	**1.2**
**Allo (0.5) + Zan (35)**	26.6	26.4	26.3	26.2	25.2	25.4	26.1	25.7
	26.3	26.3	26.4	26.2	25.5	24.6	24.6	25.3
	26.9	26.2	25.9	24.8	24.1	25.6	23.6	25.1
**AVERAGE**	**26.6**	**26.3**	**26.2**	**25.7**	**24.9**	**25.2**	**24.8**	**25.4**
**SD**	**0.3**	**0.1**	**0.3**	**0.8**	**0.7**	**0.5**	**1.3**	**0.3**
**Allo (0.5) + Zan (70)**	27.9	26.0	25.6	27.0	25.4	25.5	22.5	28.1
	26.1	27.4	26.5	27.0	27.0	25.9	25.4	26.1
	26.8	25.5	27.2	25.7	27.2	26.2	22.7	23.5
**AVERAGE**	**26.9**	**26.3**	**26.4**	**26.6**	**26.5**	**25.9**	**23.5**	**25.9**
**SD**	**0.5**	**1.3**	**0.5**	**0.9**	**0.1**	**0.2**	**1.9**	**1.8**
**Allo (1) + Zan (35)**	26.3	26.3	26.4	26.2	25.5	24.6	24.6	25.3
	26.9	26.2	25.9	24.8	24.1	25.6	23.6	25.1
	27.2	26.5	26.1	26.0	24.8	25.3	24.1	24.9
**AVERAGE**	**26.8**	**26.3**	**26.1**	**25.7**	**24.8**	**25.2**	**24.1**	**25.1**
**SD**	**0.5**	**0.2**	**0.3**	**0.8**	**0.7**	**0.5**	**0.5**	**0.2**
**Allo (1) + Zan (70)**	26.8	24.6	25.6	26.0	25.8	28.4	25.6	26.0
	27.3	27.4	26.5	27.0	27.0	25.9	25.4	26.1
	26.4	25.5	27.2	25.7	27.2	26.2	22.7	23.5
**AVERAGE**	**26.8**	**25.8**	**26.4**	**26.2**	**26.7**	**26.8**	**24.6**	**25.2**
**SD**	**0.6**	**1.3**	**0.5**	**0.9**	**0.1**	**0.2**	**1.9**	**1.8**

Figure S3

A titration of the doses of Alloferon and Zanamivir drug of mice infected with influenza A virus/H1N1.
The body weight curves of animals following the intranasal administration of alloferon and zanamivir. There were ten test groups, with the following regimens: (1) vehicle (2) virus only (3) virus + alloferon 0.5 μg/mL (4) virus + alloferon 1 μg/mL (5) virus + zanamivir 35 μg/mL (6) virus + zanamivir 70 μg/mL (7) virus + alloferon 0.5 μg/mL + zanamivir 35 μg/mL (8) virus + alloferon 0.5 μg/mL + zanamivir 70 μg/mL (9) virus + alloferon 1 μg/mL + zanamivir 35 μg/mL (10) virus + alloferon 1 μg/mL + zanamivir 70 μg/mL, administered daily for 14 days.

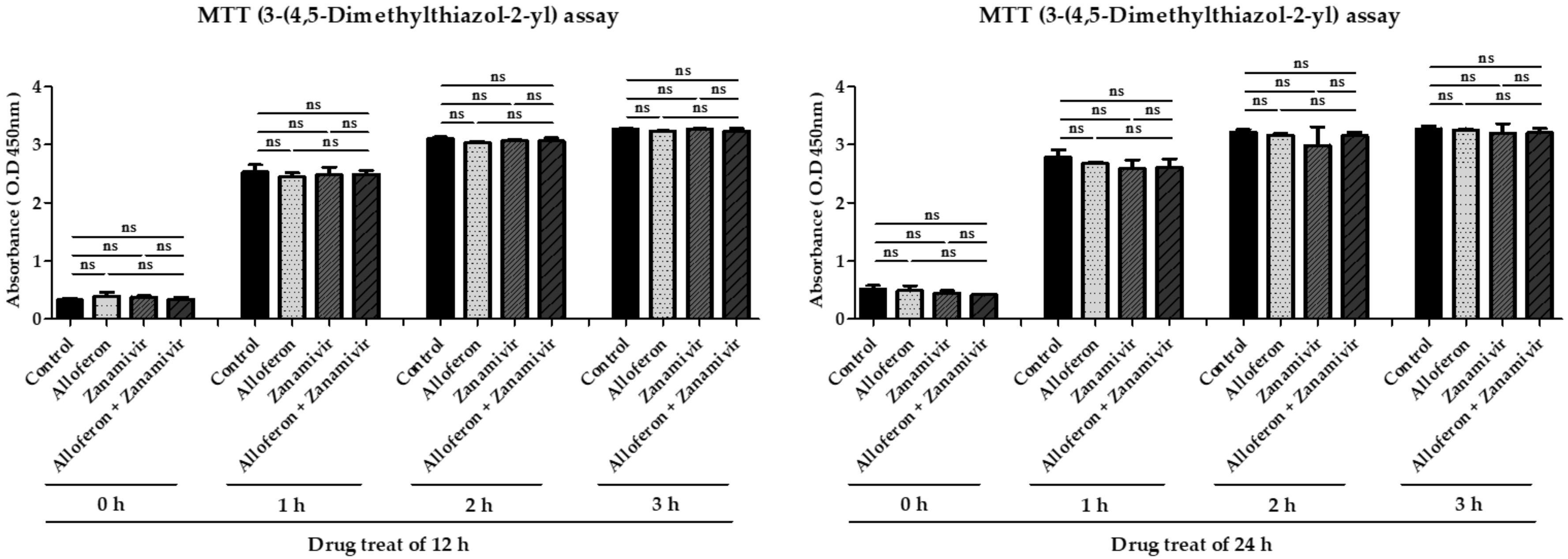

Figure S4

MTT (3-(4,5-dimethylthiazol-2-yl)-2,5-diphenyl-2H-tetrazolium bromide) assay to evaluate the cytotoxic potential of drug, alloferon and zanamivir.
An MTT assay was used to investigate the cytotoxicity of both alloferon and zanamivir. MTT assay showing cell viability on 12 hrs and 24 hrs after adding concentrations of alloferon (0.5 μg/mL) and zanamivir (35 μg/mL). The cells were incubated with the MTT reagent for 0 h, 1 h, 2 h and 3 h and the absorbance of the samples was assessed after each time period. The data represents the mean of triplicate wells of 96-well plates with standard deviation (SD) represented by error bars. NS; No Significant.

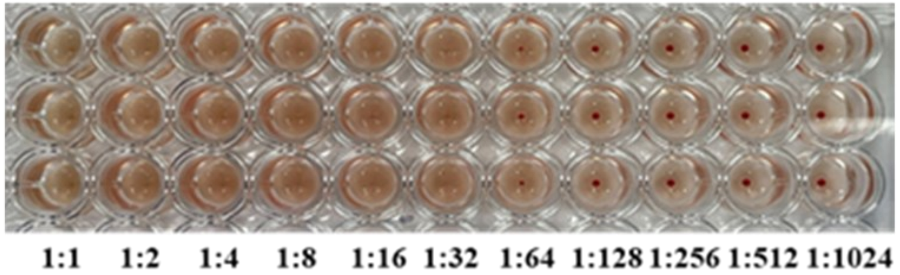

Figure S5

Hemagglutination assay for influenza A virus/H1N1 titer determination.

Hemagglutination assays can be used to detect infectious diseases such as influenza, adenovirus, and rabies. A standardized concentration of RBCs is added to each well after virus serial dilutions. At room temperature, the plate is incubated for 30 minutes. Upon completion of the incubation period, the assay can be analyzed to distinguish between agglutinated and non-agglutinated wells. Virus titers are determined from the well with the last agglutinated appearance, immediately prior to the appearance of a pellet. Based on the dilution method, the virus concentration was diluted to saline at a ratio of 1:32.

A

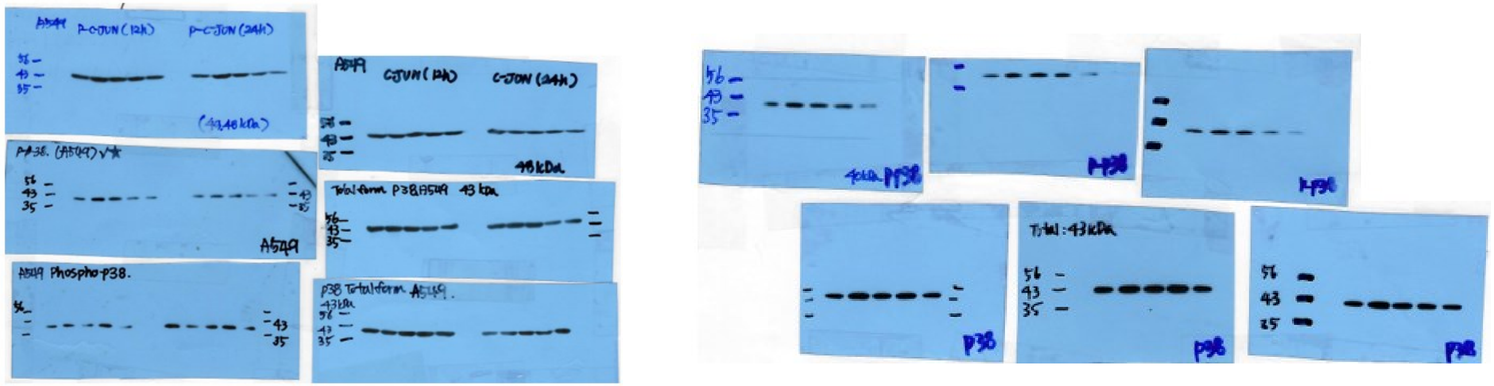

B

A549		Phospho form						Total form						Phospho form/Total form		
p38	Control	9585.832	1	11406.15	1	10084.95	1	27807.08	1	28013.9	1	27391.54		0.344726	0.40716	0.368178
12h	H1N1	24934.13	2	24800.71	2	24983.42	2	31637.15	2	31434.68	2	31184.44		0.788128	0.78896	0.80115
	Alloferon	21080.71	3	17510.49	3	20823.59	3	29427.37	3	28448	3	27625.66		0.716364	0.736808	0.753777
	Zanamviir	17427.66	4	20960.71	4	17697.9	4	26417.08	4	26434.66	4	27056.61		0.659712	0.662406	0.654106
	Allo+Zana	15282.42	5	16342.2	5	15004.35	5	29779.44	5	30884.73	5	29105.92		0.513187	0.529135	0.515508
24h	Control	7521.468	1	7416.104	1	7470.225	1	32856.46	1	33252.34	1	33539.46		0.228919	0.223025	0.222729
	H1N1	19461.13	2	19612.78	2	19392.71	2	27578.56	2	27432.85	2	27046.85		0.705661	0.714938	0.717004
	Alloferon	19083.2	3	19097.9	3	19035.66	3	16768.71	3	16803.05	3	16975		1.138024	1.136573	1.121394
	Zanamviir	25011.15	4	24909.87	4	25159.27	4	17685.66	4	17436.54	4	17349.37		1.414205	1.428602	1.450155
	Allo+Zana	7344.61	5	7385.439	5	7358.731	5	22247.56	5	22207.97	5	22226.97		0.330131	0.332558	0.331072

Mouse		Phospho form						Total form						Phospho form/Total form		
p38	Control	20196.49	1	20772.9	1	20848.02	1	20398.02	1	20545.97	1	20444.44		0.8231	0.793	0.80908
	H1N1	27941.39	2	27631.27	2	27837.97	2	27799.78	2	28043.44	2	27459.54		1.005094	0.985302	1.013782
	Alloferon	21605.68	3	21105.08	3	21613.56	3	25332.54	3	24947.13	3	25819.25		0.852883	0.845992	0.83711
	Zanamviir	11627.49	4	11974.85	4	12105.85	4	30348	4	30112.35	4	30061.64		0.383139	0.397673	0.402701
	Allo+Zana	7077.66	5	6126.175	5	6696.589	5	19951.56	5	19872.13	5	20028.85		0.354742	0.30828	0.334347

Figure S6

Image J analysis of p38MAPK results for A549 cells and Mice.

In the group treated only with Zanamivir, P38MAPK phosphorylated levels are decreased, but the total levels of P38MAPK also decrease, which seems to indicate that P38MAPK is also decreased. Therefore, by dividing phosphorylated p38MAPK by total p38MAPK, there is no difference in the ratio. We analyzed all of the bands of p38MAPK using Image J software and Excel raw data to clarify the results. [A: Western blotting band, raw data B: Image J analysis, raw excel data]
